# Assessing the impact of small firm dynamics on public mental health amid the pandemic in Latin America

**DOI:** 10.1186/s12889-024-19341-9

**Published:** 2024-07-10

**Authors:** Fernando Díaz, Pablo A. Henríquez

**Affiliations:** 1 Departamento de Ingeniería Comercial, Universidad Técnica Federico Santa María, Av. Santa María 6400, Santiago, Región Metropolitana 7630000 Chile; 2 Departamento de Administración, Universidad Diego Portales, Av. Sta. Clara 797, Santiago, 8581169 Chile

**Keywords:** Well-being, Small business, Google Trends, Facebook Business Activity

## Abstract

**Objective:**

The aim of our study is to examine the relationship between the economic activity of small firms and the mental well-being of the population in five Latin American countries in the early stages of the pandemic.

**Methods:**

We utilize the search volume of certain keywords on Google Trends (GT), such as “boredom,” “frustration,” “loneliness,” “sleep”, “anxiety”, and “depression”, as an indicator of the well-being of the population. By examining the data from Facebook Business Activity Trends, we investigate how social attention reacts to the activity levels of different economic sectors.

**Results:**

Increased business activity is generally associated with reduced levels of boredom, loneliness, sleep problems and anxiety. The effect on depression varies by sector, with positive associations concentrated in onsite jobs. In addition, we observe that strict Non-Pharmaceutical Interventions (NPIs) tend to exacerbate feelings of boredom and loneliness, sleep issues, and anxiety.

**Conclusions:**

Our findings suggest a strong association between different indicators of psychological well-being and the level of activity in different sectors of the economy. Given the essential role of small and medium-sized enterprises (SMEs) in generating employment, especially during crises like the pandemic, it is imperative that they remain resilient and adaptable to support economic recovery and job preservation. To accomplish this, policymakers need to focus on providing financial stability and support for SMEs, fostering social support networks within companies, and incorporating mental health services into workplace environments. This comprehensive strategy can alleviate mental health challenges and enhance public health resilience.

## Introduction

The COVID-19 pandemic, caused by the SARS-CoV-2 virus, has profoundly influenced numerous facets of life worldwide, affecting health, economies, social structures, and the environment. In terms of health consequences, COVID-19 has triggered major crises globally, straining healthcare infrastructures and resulting in substantial death tolls. By December 2023, it had taken the lives of almost seven million people around the world^1^.

Notably, Latin America, which comprises just 8% of the global population, represented more than 30% of worldwide COVID-19 fatalities by December 2021 [1]. Significant levels of anxiety and depression were observed due to fear of COVID-19 and socioeconomic disruptions [2, 3]. Additionally, the mortality rate for noncommunicable diseases increased by 15% due to interruptions in health services, accompanied by a decline in hospitalizations [4].

Considering other aspects of the crisis, the socioeconomic traits of Latin American nations meant that school closures impacted the development of human capital, particularly for children from underprivileged backgrounds, possibly exacerbating long-term inequality of opportunity [5]. Finally, there could be enduring environmental consequences. Although a temporary decline in air pollution has been noted due to reduced industrial operations, policymakers must also address worries about rising deforestation and other environmental challenges [6].

The COVID-19 pandemic, has caused a significant upheaval in global economies, especially impacting small enterprises and low-income countries [7, 8]. The pandemic’s social distancing measures have particularly affected the service industry, which relies on close physical interaction and is dominated by smaller businesses. Various economic sectors experienced different impacts, with smaller businesses and self-employed individuals facing more challenges than larger firms, due to differences in financial resilience, operational adaptability, and resource availability [9].

During crises, financial resilience distinguishes small from large businesses. Small businesses and self-employed individuals, often with limited cash reserves and financial safety nets, are more vulnerable to economic downturns [10, 11]. They also struggle with adaptability, particularly in transitioning to digital platforms due to limited technological infrastructure [9]. In contrast, large businesses, equipped with better technological resources, adapt more readily to digital operations [12]. Additionally, small businesses frequently face challenges in accessing government support, which tends to favor large businesses with stronger banking ties and better administrative capacity [11, 13, 14].

Economic uncertainty and insecure job conditions are significant contributors to mental health concerns. Recent studies during the COVID-19 pandemic have shown a clear association between job loss, economic shocks, and mental health. Evidence from multi-country analyses indicates that such economic disruptions are potent stressors affecting mental health outcomes [15, 16].

In this article, we analyze the impact of local business economic activity on the mental health of the population in Argentina, Chile, Colombia, Peru, and Mexico. The level of dynamism of various business activities is proxied by the Business Activity Trends (BAT) data from Meta. We follow [2, 17, 18] and use Google Trends (GT) data to proxy for changes in the mental well-being of the population. Our findings indicate a negative correlation between the activity levels of local businesses in various sectors of the economy and the public interest in topics related to psychological well-being, such as boredom, loneliness, sleep, and anxiety. This relationship remains consistent even after controlling for country time-invariant fixed effects, the stringency of nonpharmaceutical interventions, and measures of overall economic conditions.

Due to the wide presence of small businesses on the Facebook platform, the use of BAT measures is particularly suitable for assessing the impact of economic uncertainty on the psychological well-being of the population of our sample countries, since small businesses absorb a large fraction of jobs in these countries^2^.

Our research is in line with studies like [19, 20], which investigate the impact of the pandemic on small businesses, urban mobility, and mental health in the USA and Latin America. These studies show significant adverse effects on employment, especially for smaller enterprises. Most related literature, using surveys and online questionnaires, examines pandemic effects on public welfare [21–23]. While useful, this approach lacks pre-pandemic data, limiting the understanding of the pandemic’s full impact [18]. A solution is using historical Google Trends (GT) data, which, despite losing demographic details, provides reliable insights into population well-being through search behaviors [24–28]

This study makes a significant contribution to the field of social media mining research by offering a novel analysis of the influence of local and small business economic activities on public emotions. By leveraging data from Google Trends to gauge social attention and utilizing Facebook Business Activity Trends to monitor the dynamics of small business operations, it provides a unique perspective on how variations in the economic activities of small and medium-sized enterprises (SMEs) can impact community well-being. This approach not only advances our understanding of the interplay between economic activity and public sentiment, but also underscores the importance of SMEs in the economic fabric of societies, particularly through the lens of social media analytics.

## Methods

### Sample

We consider information for Business Activity Trends for Argentina, Chile, Colombia, México, and Perú, for the early months of the COVID-19 pandemic, from March 1, 2020, to August 29, 2020. For this sample period, we obtained daily online search data from GT on the following topics related to population wellness: *Boredom, Loneliness, Sleep, Depression, Anxiety* and *Frustration*. To obtain a proper measure of social attention towards these topics, a baseline measure, under normal, non-pandemic conditions is required. Therefore, we obtained online search data for the analogous period during 2019.

The countries included in this study-Argentina, Chile, Colombia, Mexico, and Peru-share similar socioeconomic and public health challenges, such as significant economic disparities and constrained healthcare access. These factors make them suitable for analyzing the impact of economic activities on mental health. Additionally, the uniform use of spanish facilitates consistent data collection and analysis using Spanish keywords in Google Trends, ensuring comparability across these nations^3^.

### Social attention to mental health problems and psychological distress

Following [2, 29], our proxies for the population’s attention towards mental health disorders are based on GT, a service that shows the frequency of worldwide searches [30, 31]. GT produces time series data on *Search Volume Intensity* (SVI) to measure social attention to a particular keyword in a specific period and location [32]. The SVI is measured on a scale of 0 to 100, where 0 represents complete disinterest and 100 represents the highest level of popularity. During the process of data collection, values below 1, denoted as $<1$ by GT, were replaced by 1.

We obtained the daily volume of Google searches for the topics presented in Table 1, translated into Spanish. These terms refer to standard indicators that measure the emotional effects caused on the population by both the COVID-19 pandemic and the containment policies implemented by governments in response to the disease. We refer to recent work using these terms in the last column of this table.

**Table 1:** Keywords used for searches in Spanish on Google Trends, their corresponding translations, and recent literature in which they appear

Topic	Keywords (Searches in Spanish)	Keywords in English	Article
*Boredom*	Aburrimiento, Tedio, Fastidio, Aburrido, Aburrida, Qué lata, Qué fome	Monotony, Dullness, Doldrums, Tedium, Tiresomeness, Wearisomeness	[18, 29]
*Loneliness*	Soledad, Aislamiento, Solo, Sola, Abandono, Incomunicación, Incomunicado, Separación, Quiebre, Ausencia, Encierro, Encerrado, Encerrada	Solitude, Isolation, Lonesomeness, Separation, Solitariness, Loneliness, Alienation, Friendlessness, Lonely Feeling, Feeling Alone	[18, 29, 33]
*Sleep*	Dormir, Dormir bien, Insomnio, Desvelo, Devalado, Desvelada	Snooze, Rest, Doze, Repose, Siesta, Nap, Catnap, Hibernation	[18, 29, 34, 35]
*Frustration*	Frustración, Frustrado, Frustrada, Impotencia, Apestado	Annoyance, Anger, Resentment, Disappointment, Discomfiture, Dismay, Chagrin, Dissatisfaction, Detdown	[29, 36]
*Depression*	Depresión, Decaimiento, Desánimo	Depression, Decay, Discouragement	[2, 19]
*Anxiety*	Ansioso, Ansiosa, Angustia, Inquietud, Preocupación	Anxious, Distress, Restlessness, Worry	[19, 37]

In order to study how changing patterns in the different verticals of the BAT may affect the social attention to the keywords presented in Table 1, we compute an *Abnormal Search Volume Activity* index, $ASVA_{s,t}$:

1 $$\begin{aligned} ASVA_{s,t}=ln \left( \frac{SVI_{s,t}}{E(SVI^*_{s,t})} \right) \end{aligned}$$

where *ln* denotes natural logarithm and the $E(SVI^*_{s,t})$ is computed as the monthly average of the SVI index for keywords during the corresponding month in 2019 [2, 29, 38].

The $SVI_{s,t}$ for each keyword during the pandemic months (March to August 2020) was compared against the same months in 2019 ($SVI^*_{s,t}$). This comparison allowed us to identify significant deviations in public interest and concern, which are indicative of the broader mental health impacts of the pandemic. By analyzing these differences, our study not only highlights the direct effects of COVID-19 on mental well-being but also underscores the importance of accessible mental health resources during unprecedented public health crises. This analysis is central to our study, demonstrating how societal behavior, reflected through online searches, can provide critical insights into the mental health landscape during times of global distress.

Table 2 displays the descriptive statistics for the entire sample of the ASVA of the topics outlined in Table 1.

**Table 2:** ASVA Descriptive Statistics for Key Terms in Table 1 aggregated across countries

Statistic	Mean	Median	Pctl(25)	Pctl(75)	St. Dev.
Boredom	0.14	0.17	−0.13	0.43	0.44
Frustration	−0.11	0.03	−0.46	0.38	0.79
Loneliness	0.05	0.06	−0.09	0.18	0.20
Sleep	−0.02	−0.01	−0.22	0.22	0.35
Anxiety	0.12	0.17	−0.15	0.47	0.56
Depression	−0.08	−0.05	−0.36	0.25	0.54

### Facebook Business Activity Trends

Business Activity Trends gather data from Facebook Business Pages to measure the variation in activity among local businesses globally and their response and recovery to crises over time. Essentially, these trends are determined by analyzing the rate at which businesses post on Facebook. Business verticals are derived by aggregating categories as defined by the administrators on the corresponding business pages. Business verticals, as defined by *Data for Good at Meta* [39, 40], are shown in Table 3,^4^.

Grocery and convenience stores es GCS, Retai es Ret, Restaurants es Rest, Local events es Local, Professional services es Profe.Serv, Business and utility services es Bus, Home services es Home, Lifestyle services es Lifestyle, Travel es Trav, Manufacturing es MFG, Public good es Pub

**Table 3:** Definitions for Business Verticals as defined by Meta

Business Vertical	Description
**All**	Refers to all businesses in the polygon.
**Grocery and convenience stores**	Retailers that sell everyday consumable goods including food (typically unprepared foods and ingredients) and a limited range of household goods (like toilet paper). These can include grocery stores, convenience stores, pharmacies and general stores.
**Retail**	Retail other than grocery and convenience stores such as auto dealers, home goods stores, personal goods stores and general merchandise/big-box stores like Walmart.
**Restaurants**	Businesses that sell prepared food and beverages for on-premise or off-premise dining.
**Local events**	Events, activities and businesses that sell real-life experiences, such as amusement parks, bowling alleys, concert venues and social clubs.
**Professional services**	Services driven by demand from an individual event such as a legal need or health issue that require high customization. Providers usually have an advanced degree or certification and are considered experts and “knowledge workers.” Examples include CPAs, lawyers, medical professionals, architects.
**Business and utility services**	Business services offering business-to-business services like construction, office cleaning, advertising and marketing companies and business software solutions. Utility services offer commodity services like electric, phone, internet, water and energy.
**Home services**	Services driven by demand from an individual event at home such as plumbing or electrical work. Examples include home repairs, photographers, cleaning, mechanics, plumbers, electricians, landscapers, interior decorators.
**Lifestyle services**	Specific to beauty, care and fitness services. These businesses offer standardized services that are part of a customer’s regular routines. Examples include gyms, salons, barbers, and nonmedical and noneducational supervision, like childcare nurseries and pet care.
**Travel**	Businesses that provide or sell transportation or accommodation services, such as airlines, hotels, car rentals and tour operators.
**Manufacturing**	Businesses that manufacture durable goods (like furniture and cars) or consumable goods (like food and personal goods) and have no or limited business-to-customer sales .
**Public good**	Includes government agencies, nonprofits and religious organizations.

**Fig. 1:**
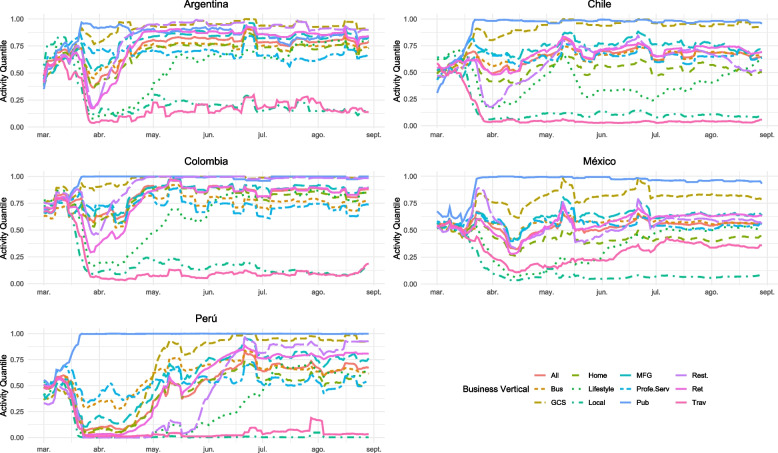
A **Business Activity Trends.** GADM0. The level of activity as a quantile relative to the baseline period, corresponding to the pre COVID 19 pandemic crisis. The baseline period is used to represent “normal” daily business activities in the absence of a crisis

Figure 1 illustrates the evolution of the Activity Quantiles of the Business Activity Trends for the countries included in our sample^5^. There is considerable variation in the levels of activity observed in different business verticals, both within a country and when comparing different countries within the same sector.

In examining the trends depicted in Fig. 1, notable variations in business activity levels are evident among the countries studied. Argentina and Colombia display similar patterns, likely reflective of analogous economic structures and governmental pandemic responses that prioritize certain industries. Chile and Mexico, meanwhile, exhibit parallel trends, potentially due to their more diversified economies and robust public health infrastructures, which may have enabled a more resilient business response during the pandemic. In contrast, Peru shows a distinctive trajectory, possibly influenced by its unique challenges such as political instability and inconsistent public health measures during the same period. These differences highlight the complex interplay between national economic policies, public health infrastructure, and the resilience of business activities, underscoring the nuanced impacts of the pandemic across different Latin American contexts.

Table 4 displays the descriptive statistics for the entire sample of the Activity Quantiles for the Business Verticals in Table 3, aggregated across countries.

**Table 4:** Descriptive statistics for activity quantiles aggregated across countries

Statistic	Mean	Median	Pctl(25)	Pctl(75)	St. Dev.
All	0.65	0.67	0.56	0.79	0.18
Grocery & convenience stores	0.84	0.93	0.82	0.97	0.21
Retail	0.67	0.71	0.55	0.84	0.21
Restaurants	0.68	0.71	0.51	0.92	0.27
Local Events	0.16	0.10	0.06	0.18	0.18
Professional Services	0.63	0.64	0.55	0.70	0.10
Business & utility services	0.65	0.66	0.58	0.74	0.11
Home Services	0.58	0.56	0.45	0.74	0.19
Lifestyle Services	0.46	0.50	0.26	0.67	0.25
Travel	0.17	0.10	0.04	0.23	0.17
Manufacturing	0.71	0.74	0.63	0.84	0.17
Public Good	0.93	0.98	0.92	1.00	0.11

### Estimation

We consider a panel data specification where we regress the ASVA for each of the keywords in Table 1 against the activity quantities of each of the business verticals.

2 $$\begin{aligned} ASVA_{it}^{j}=\alpha _{i} + \beta _1 Activity_{it}^{k} + \beta _2 Str\_Index_{it} + \mu _{it}, \end{aligned}$$

where $ASVA_{it}^{j}$ is the *Abnormal Search Volume Activity* index for keyword *j* in country *i* at time *t*, $Activity_{it}^{k}$ is the activity quantile for business vertical *k* in country *i* at time *t*, $Str\_Index_{it}$ is the *Stringency Index* (SI) for country *i* at time *t*, and $\alpha _i$ corresponds to country fixed effects. As usual, $\mu _{it}$ is the error term for country *i* at time *t*. The SI quantifies the stringency of government responses to the pandemic in a composite measure ranging from 0 to 100, where a higher score indicates more stringent policies [41]. The index has been used to evaluate the influence of COVID-19 policies on economic activities [42, 43], in the realm of public health, to examine the efficacy of *Non-Pharmaceutical Interventions* (NPI) [44, 45], and their effect on the mental well-being of the population [46, 47]. In this context, it is a suitable control to evaluate the influence of the varying levels of dynamism in the various business activities being examined on the level of societal focus on mental health-related topics.

## Results

### Main results

In this section, we examine the relation between the ASVA of the keywords specified in Table 1 and the levels of economic activity in the business verticals, taking into account country fixed effects and the SI within each country. Table 5 presents the results of estimating Eq. (2). Panel A reports the coefficients for the level of activity of the various verticals, while Panel B presents the estimations for the coefficients of the SI. Panel C includes some basic regression statistics.

**Boredom**: For *Boredom*, when considering all business verticals in column (1), the coefficient for the level of activity of the corresponding vertical is found to be negative and statistically significant at the 1% level or higher. Also, the economic significance is substantial; an increase of one standard deviation (0.18) in the activity of local businesses results in a decrease of 0.084 ($-0.464 \cdot 0.18$) in boredom’s ASVA.The reduction exceeds half of the mean sample value for boredom in our dataset (0.14) and is 0.19 times its standard deviation. In general, the estimated coefficients for the activity levels of the remaining columns (2 to 12) mostly exhibit negative values, with the majority of them being statistically significant at the 1% level. These results indicate that a rise in the economic activities of firms across various sectors is associated with lower levels of boredom among the population.The estimated coefficients for the business sectors *Professional Services* and *Public Good* stand out as they are both positive (and significantly different from zero). Furthermore, most of the estimated coefficients associated with the SI in Panel B are positive, indicating that the adoption of stricter measures to address the pandemic results in higher levels of boredom among the population.

**Loneliness**: With respect to *Loneliness*, in most sectors the coefficient for *Activity* is negative and statistically significant, with large economic effects. For example, in the case of *Lifestyle Services*, an increase of one standard deviation (0.25) in economic activity results in a decrease of 0.03 ($-0.137 \cdot 0.25$) in the ASVA of loneliness, a rather large value corresponding to almost 8% of its sample mean value. Therefore, an increase in economic activity is linked to a decrease in public emotions associated with loneliness. It is worth noting that the estimated coefficients for the business verticals “Professional Services” and “Public Good” continue to be positive and significantly different from zero. All statistically significant estimated coefficients of the SI are positive, indicating that the implementation of stricter NPI measures leads to increased levels of loneliness in the population.

**Sleep**: For the ASVA of *Sleep*, the estimated coefficients for the activity of all business verticals are negative and almost all result statistically and economically significant; for example, an increase of one standard deviation (0.18) in the activity of all local businesses results in a decrease of 0.126 ($-0.702\cdot 0.18$) in the ASVA for sleep, which is more than six times the sample mean of this variable (-0.02) and more than one third of its sample standard deviation (0.35). Furthermore, as expected, the estimated coefficients for the degree of stringency of NPIs are positive and significantly different from zero. According to our results, an increase in the level of activity in the different sectors of the economy leads to a reduction in sleep disorders in the population.

**Frustration**: Regarding *Frustration*, our analysis suggests that the activity of local businesses does not influence the level of social attention towards this topic. Moreover, the majority of the coefficients for the SI outcome are statistically not significantly different from zero.

**Depression**: We find that public attention to *Depression* is positively associated with economic activity. For six of our business verticals -*All, Business and utility services, Grocery and convenience stores, Manufacturing, Restaurants and Retail* -, the estimated effects are positive and statistically different from zero, with large economic effects; for instance, when every business within a specific polygon is considered - *All* - an increase of one standard deviation in the activity of local businesses (0.18) results in an increase of 0.032 ($0.176 \cdot 0.18$) in depression’s ASVA, 40% of its mean value and almost 6% of its standard deviation.

**Anxiety**: We find some evidence that *Anxiety* in the population is inversely associated with increases in economic activity. Only one business vertical, *Lifestyle*, is statistically significant at the 1% level and three orders exhibit coefficients for the level of activity that are negative and significantly different from zero at the 10% level. Regardless, when statistically significant, the estimated effects are economically important. For example, for * Business and utility services*, an increase of one standard deviation (0.11) in economic activity results in a decrease of 0.0436 ($-0.396 \cdot 0.11$) in the ASVA of loneliness, a value that corresponds to 21% of its sample standard deviation and more than one third of its sample average.

Regarding the SI, as shown in Panel B of Table 5, for most of the business verticals and keywords in Table 1, the estimated coefficients tend to be positive, and for *Boredom*, *Sleep*, and *Loneliness*, also highly statistically significant. These results are consistent with the findings in previous studies that suggest that higher policy stringency is associated with higher psychological distress [46–49].

Interestingly, the effect of the SI on *Depression* is negative, which is the opposite of the expected sign. The World Health Organization (WHO) highlights depression as a major global health concern, especially with the ongoing Covid-19 pandemic. According to the WHO, over 280 million people worldwide suffer from depression, a common mental disorder. Women are more frequently impacted than men, and the disorder can severely hinder daily activities, work, and social bonds. The pandemic has aggravated the situation, causing a 25% rise in global anxiety and depression rates within the first year^6^. For instance, [50] find that social isolation -which is positively associated with the SI-and health-related fears, have contributed to rising depression rates of frontline health care workers. With respect to specific demographics, [51] find that young adults and women have been particularly affected. Several studies highlight the role of prolonged stress in exacerbating depressive symptoms [52–54]. Nevertheless, the fact that the level of the SI on *Depression* is negative aligns with our result that this phenomenon is linked to the level of engagement in certain economic industries. In other words, our findings imply that there is a trade-off between the chance of securing employment, which is closely linked to increased economic activity, and the likelihood of contracting the disease.

**Table 5:** Estimation results for Eq. (2). The number of country-day observations in accordance with the particular specification ranges from 905 to 910. All specifications include country fixed-effects

	Business Vertical											
Topic	All	Bus	GCS	Home	Lifestyle	Local	MFG	Prof. Serv	Pub	Rest	Ret	Trav
	(1)	(2)	(3)	(4)	(5)	(6)	(7)	(8)	(9)	(10)	(11)	(12)
	Business Activity											
Boredom	-0,464***	-0.616***	-0.202**	-0.393***	-0.580***	-0.064	-0.402***	0.789***	1.325***	-0.282***	-0.453***	-0.847***
	(0.11)	(0.161)	(0.085)	(0.123)	(0.070)	(0.228)	(0.107)	(0.218)	(0.300)	(0.065)	(0.076)	(0.191)
Loneliness	-0.046	-0.126*	0.039	-0.035	-0.137***	-0.311***	-0.006	0.246***	0.703***	-0.0003	-0.093***	-0.392***
	(0.044)	(0.064)	(0.034)	(0.049)	(0.028)	(0.089)	(0.043)	(0.087)	(0.118)	(0.026)	(0.031)	(0.075)
Sleep	-0.702***	-0.920***	-0.446***	-0.778***	-0.519***	-0.065	-0.617***	-0.923***	-0.145	-0.324***	-0.465***	-0.643***
	(0.071)	(0.106)	(0.056)	(0.079)	(0.046)	(0.154)	(0.070)	(0.145)	(0.205)	(0.043)	(0.050)	(0.129)
Frustration	0.229	0.449	0.142	0.299	-0.012	-0.268	0.241	0.182	0.469	0.124	0.200	-0.602*
	(0.197)	(0.288)	(0.151)	(0.219)	(0.129)	(0.404)	(0.191)	(0.390)	(0.539)	(0.117)	(0.138)	(0.342)
Depression	0.176**	0.229*	0.234***	0.154	0.045	-0.060	0.246***	0.027	0.331	0.106**	0.140**	-0.012
	(0.087)	(0.127)	(0.066)	(0.097)	(0.057)	(0.179)	(0.084)	(0.172)	(0.238)	(0.052)	(0.061)	(0.151)
Anxiety	-0.163	0.396*	-0.105	-0.261*	-0.345***	0.026	-0.205	0.076	0.044	0.018	-0.170*	-0.375
	(0.142)	(0.207)	(0.108)	(0.157)	(0.092)	(0.289)	(0.137)	(0.279)	(0.386)	(0.084)	(0.099)	(0.245)
	Stringency Index											
Boredom	0,002***	0.003***	0.003***	0,002***	0.0003	0.002	0.003***	0.002***	-0.003**	0.002***	0.002***	-0.003**
	(0.001)	(0.001)	(0.001)	(0.001)	(0.001)	(0.002)	(0.001)	(0.001)	(0.001)	(0.001)	(0.001)	(0.001)
Loneliness	0.002***	0.002***	0.002***	0.002***	0.002***	0.0001	0.002***	0.002***	-0.001	0.002***	0.002***	-0.00004
	(0.0003)	(0.0003)	(0.0003)	(0.0003)	(0.0003)	(0.001)	(0.0003)	(0.0003)	(0.001)	(0.0003)	(0.0003)	(0.001)
Sleep	0.003***	0.004***	0.005***	0.003***	0.001***	0.003**	0.004***	0.003***	0.004***	0.003***	0.003***	-0.0005
	(0.0004)	(0.0004)	(0.0005)	(0.0004)	(0.0004)	(0.001)	(0.0004)	(0.0004)	(0.001)	(0.0004)	(0.0004)	(0.001)
Frustration	0.002*	0.002	0.002	0.002**	0.002*	0.0003	0.002	0.002*	0.0001	0.002*	0.002*	-0.001
	(0.001)	(0.001)	(0.001)	(0.001)	(0.001)	(0.003)	(0.001)	(0.001)	(0.003)	(0.001)	(0.001)	(0.002)
Depression	-0.001*	-0.001**	-0.002***	-0.001	-0.001	-0.001	-0.001***	-0.001*	-0.002**	-0.001**	-0.001**	-0.001
	(0.001)	(0.001)	(0.001)	(0.001)	(0.001)	(0.001)	(0.001)	(0.001)	(0.001)	(0.001)	(0.001)	(0.001)
Anxiety	0.002**	0.002***	0.002***	0.002**	0.001	0.002	0.002***	0.002**	0.002	0.002**	0.002**	-0.0002
	(0.001)	(0.001)	(0.001)	(0.001)	(0.001)	(0.002)	(0.001)	(0.001)	(0.002)	(0.001)	(0.001)	(0.002)
	Regression Statistics (Obs, R2, F-test)											
Boredom	0.033	0.03	0.02	0.025	0.084	0.014	0.029	0.028	0.035	0.034	0.051	0.035
	15,546***	13,911***	9,415***	11,728***	41,613***	6,572***	13,704***	13,159***	16,407***	16,045***	24,373***	16,535***
Loneliness	0.080	0.082	0.080	0.079	0.102	0.091	0.078	0.087	0.113	0.078	0.088	0.105
	39.053***	40.523***	39.166***	38.724***	51.091***	45.021***	38.459***	42.809***	57.769***	38.448***	43.395***	53.201***
Sleep	0.149	0.131	0.120	0.148	0.175	0.058	0.132	0.098	0.058	0.113	0.139	0.083
	78.891***	67.793***	61.767***	78.646***	95.526***	27.654***	68.904***	48.941***	27.825***	57.383***	72.973***	40.825***
Frustration	0.005	0.006	0.005	0.006	0.004	0.004	0.006	0.004	0.005	0.005	0.006	0.007
	2.381*	2.919*	2.148	2.640*	1.708	1.925	2.504*	1.813	2.085	2.266	2.761*	3.263*
Depression	0.008	0.007	0.018	0.007	0.005	0.004	0.013	0.004	0.006	0.009	0.010	0.004
	3.794**	3.376**	8.023***	3.023**	2.066	1.809	6.044***	1.766	2.723*	3.862**	4.396**	1.756
Anxiety	0.008	0.010	0.007	0.009	0.022	0.006	0.009	0.006	0.006	0.006	0.010	0.009
	3.542**	4.716***	3.348**	4.266**	10.033***	2.878*	3.999**	2.911*	2.880*	2.897*	4.358**	4.058**

Statistically significant at $$^{*}p<$$0.1; $$^{**}p<$$0.05; $$^{***}p<$$0.01

### Robustness check: controlling for economic wide activity indicators

So far, we have analyzed the impact of small business dynamism, as measured by the *Business Activity Trends* data from Meta, on the psychological well-being of the population in our sample countries. Our results suggest that there is an inverse relationship between the level of economic activity of SMEs in different economic sectors and the amount of social attention given to search topics related to well-being, in particular to *Boredom*, *Sleep*, and *Loneliness*.

However, one could argue that the observed effect is a result of the impact of general economic activity on the well-being of the population, rather than that of the dynamism of smaller firms captured by BAT. In order to tackle this issue, we incorporate a proxy that represents the sectoral level of economic activity as reported by the countries in our sample within the empirical model specified in Eq. (2).

Official estimates of economic activity in various sectors, as reported by economic authorities, are generally available on a monthly basis. For example, in Chile, the **IMACEC**, known as the Monthly Economic Activity Index, serves as a proxy for the performance of various economic sectors within a specific month. The computation of the Monthly Economic Activity Index relies on several supply metrics, which are adjusted based on the contribution of different economic sectors to the GDP of the previous year^7^$$^,$$^8^. Nonetheless, there are no exact correspondences between the categories established by Meta for their business verticals and the sector categorizations in the economic indices offered by the authorities, which also differ across countries. Therefore, to properly control for sectoral economic activity in specification (2), in Table 6 we match, for each country, the most similar economic sector to each of the verticals specified by Meta. Although the match is not perfect, based on our understanding, it is sufficiently close to achieve the intended control in our empirical specifications. In Table 7, we present, for each country, the correlation between the verticals of the BAT provided by Meta and the official countries’ sectoral economic activity indices^9^. Interestingly, correlations, although not particularly large in absolute value, tend to be negative and statistically significant. This suggests that SMEs may rely on non-traditional channels, such as social media, to improve their business during economic downturns.

In Table 8 we present the results of the estimation of Eq. (2) when the level of the sector economic index corresponding to the matched vertical is included as a control in our empirical specifications. In general, the findings align closely with our previous analyses. The point estimates for the coefficients for the level of activity of the various verticals in Panel A have the same signs and are of the same order of magnitude as those presented in Table 5. In addition, the levels of significance are essentially unchanged, with significant effects concentrated on *Boredom*, *Sleep*, and *Loneliness*. Also, for these topics, the effects of the SI shown in Panel B are positive and significantly different from zero, similar to those reported in Table 5.

In Panel C of Table 8 we present the estimates for the effect of the officially reported economic activities on the ASVA of our search topics. For most of our search terms and business verticals, these effects are statistically insignificant. However, for social attention towards *Sleep*, all the point estimates are negative and statistically significant at the 1% level. For *Frustration*, when statistically different from zero, the effect tends to be negative. These results suggest that the level of sectoral economic activity, as officially reported by the economic authorities of our sample countries, does have an impact on certain dimensions of population well-being^10^. Finally, Panel D includes some basic regression statistics.

A significant conclusion of our research is that the functioning of local enterprises affects the community’s mental health in ways that are not entirely captured by the activity metrics of formal sector businesses, as indicated by official economic activity indices from local agencies. This suggests that SMEs employ a segment of the workforce that is not integrated by larger, more formal companies. Therefore, it is essential for policymakers to focus on providing financial support and stability for SMEs, and to foster social support networks within these businesses, to not only stimulate the economy but also to improve mental well-being during times of crisis^11^.

**Table 6:** Match between Business Verticals, as defined by Meta, and sectoral economic activity indexes reported by sample countries

**Business Vertical**	**Matched index for Argentina**
All	Original Index
Grocery	Wholesale, retail and repairs
Retail	Wholesale, retail and repairs
Restaurants	Hotels & Restaurants
Local	Other personal services activities
Prof Services	Other personal services activities
Bus and Ut services	Electricity, gas, water
Home Services	Electricity, gas, water
Lifestyle	Other personal services activities
Travel	Hotels & Restaurants
Manufacturing	Manufacturing Industry
Public Goods	Public administration and defence
**Business Vertical**	**Matched index for Chile**
All	Non mining
Grocery	Store, Shop
Retail	Retail, trade
Restaurants	Hotels & Restaurants
Local	Personal services
Prof Services	Business services
Bus and Ut services	Electricity, gas, water and waste management
Home Services	Electricity, gas, water and waste management
Lifestyle	Personal services
Travel	Hotels & Restaurants
Manufacturing	Manufacturing Industry
Public Goods	Public administration
**Business Vertical**	**Matched index for Colombia**
All	Economic Monitoring Indicator
Grocery	Wholesale and retail trade
Retail	Wholesale and retail trade
Restaurants	Commerce, accommodation and food services
Local	Arts, entertainment and recreation activities
Prof Services	Professional activities
Bus and Ut services	Electricity, gas, water and waste supply
Home Services	Electricity, gas, water and waste supply
Lifestyle	Arts, entertainment and recreation activities
Travel	Commerce, accommodation and food services
Manufacturing	Manufacturing Industry
Public Goods	Public administration
**Business Vertical**	**Matched index for Mexico**
All	All
Grocery	Wholesale and retail trade
Retail	Wholesale and retail trade
Restaurants	Food and beverage preparation services
Local	Other recreational services
Prof Services	Professional services
Bus and Ut services	Energy, water & gas
Home Services	Energy, water & gas
Lifestyle	Other recreational services
Travel	Temporary housing services
Manufacturing	Manufacturing Industry
Public Goods	Legislative activities
**Business Vertical**	**Matched index for Perú**
All	PBI
Grocery	Non-primary sectors
Retail	Retail trade
Restaurants	Other services
Local	Non-primary sectors
Prof Services	Other services
Bus and Ut services	Energy, water
Home Services	Energy, water
Lifestyle	Other services
Travel	Other services
Manufacturing	Manufacturing Industry
Public Goods	Other services

**Table 7:** Correlations between Business Activity Trends and sample countries’ sectoral economic activity indicators

	Business Vertical											
Topic	All	Bus	GCS	Home	Lifestyle	Local	MFG	Prof. Serv	Pub	Rest	Ret	Trav
	(1)	(2)	(3)	(4)	(5)	(6)	(7)	(8)	(9)	(10)	(11)	(12)
Argentina	-0.5392***	-0.2215	-0.5998***	-0.2515	-0.5290***	0.1658	-0.2549	-0.8010***	-0.7357***	-0.9158***	-0.3976**	0.4013**
	(0.0012)	(0.2155)	(0.0002)	(0.1580)	(0.0015)	(0.3564)	(0.1523)	(0.0000)	(0.0000)	(0.0000)	(0.0219)	(0.0206)
Chile	-0.7188***	-0.6385***	-0.2898	-0.5255***	-0.4165**	0.273	-0.3291*	-0.5474***	-0.1506	-0.9066***	-0.513***	0.4307**
	(0.0000)	(0.0001)	(0.1018)	(0.0017)	(0.0159)	(0.1242)	(0.0615)	(0.0010)	(0.4027)	(0.0000)	(0.0023)	(0.0124)
Colombia	-0.6976***	-0.8034***	-0.7135***	-0.7813***	-0.4039**	0.2386	-0.6251***	-0.6539***	-0.253	-0.7744***	-0.6423***	0.3104*
	(0.0000)	(0.0000)	(0.0000)	(0.0000)	(0.0197)	(0.1811)	(0.0001)	(0.0000)	(0.1554)	(0.0000)	(0.0001)	(0.0787)
Mexico	-0.5842***	0.6876***	-0.6106***	0.6897***	-0.4169**	0.0644	-0.666***	-0.4736***	0.0386	-0.8414***	-0.5043***	-0.3374*
	(0.0004)	(0.0000)	(0.0002)	(0.0000)	(0.0158)	(0.7217)	(0.0000)	(0.0054)	(0.8312)	(0.0000)	(0.0028)	(0.0548)
Perú	-0.1473	-0.6286***	-0.2084	-0.3685**	-0.0051	-0.1763	0.0785	-0.6554***	-0.5434***	-0.1658	0.1206	0.6257***
	(0.4133)	(0.0001)	(0.2446)	(0.0348)	(0.9777)	(0.3263)	(0.6639)	(0.0000)	(0.0000)	(0.3565)	(0.5038)	(0.0001)

Statistically significant at $$^{*}p<$$0.1; $$^{**}p<$$0.05; $$^{***}p<$$0.01

**Table 8:** Estimation results for Eq. (2) controlling for economic activity indexes. The number of country-day observations in accordance with the particular specification ranges from 905 to 910. All specifications include country fixed-effects

	Business Vertical											
Topic	All	Bus	GCS	Home	Lifestyle	Local	MFG	Prof. Serv	Pub	Rest	Ret	Trav
	(1)	(2)	(3)	(4)	(5)	(6)	(7)	(8)	(9)	(10)	(11)	(12)
	Business Activity											
Boredom	-0.567**	-0.493***	-0.234***	’-0.257**	-0.592***	-0.065	-0.411***	0.829***	1.363***	-0.283***	-0.447***	-0.895***
	(0.132)	(0.162)	(0.087)	(0.126)	(0.070)	(0.228)	(0.107)	(0.215)	(0.301)	(0.065)	(0.078)	(0.192)
Loneliness	-0.172***	-0.094	-0.009	0.004	-0.139***	-0.312***	-0.017	0.268***	0.693***	-0.001	-0.093***	-0.434***
	(0.052)	(0.065)	(0.034)	(0.050)	(0.029)	(0.090)	(0.043)	(0.084)	(0.118)	(0.026)	(0.031)	(0.075)
Sleep	-0.448***	-0.752***	-0.392***	-0.604***	-0.498***	-0.055	-0.570**	-0.941***	-0.056	-0.320***	-0.392***	-0.497***
	(0.084)	(0.102)	(0.057)	(0.079)	(0.046)	(0.152)	(0.069)	(0.145)	(0.203)	(0.041)	(0.049)	(0.125)
Frustration	0.476**	0.553*	0.181	0.423*	-0.041	-0.278	0.250	0.179	0.564	0.125	0.227	-0.567
	(0.236)	(0.292)	(0.155)	(0.226)	(0.129)	(0.404)	(0.192)	(0.391)	(0.540)	(0.117)	(0.140)	(0.345)
Depression	0.080	0.214*	0.216***	0.139	0.052	-0.058	0.246***	0.034	0.285	0.106**	0.148**	0.002
	(0.104)	(0.129)	(0.068)	(0.100)	(0.057)	(0.179)	(0.085)	(0.173)	(0.239)	(0.052)	(0.062)	(0.153)
Anxiety	-0.078	-0.356*	-0.103	-0.219	-0.336***	-0.031	-0.175	0.075	0.032	0.021	-0.137	-0.311
	(0.170)	(0.210)	(0.111)	(0.162)	(0.092)	(0.289)	(0.138)	(0.280)	(0.387)	(0.084)	(0.101)	(0.247)
	Stringency Index											
Boredom	0.003***	0.002***	0.004***	0.001**	0.001	0.002	0.003***	0.004***	0.004**	0.003***	0.002***	-0.002
	(0.001)	(0.001)	(0.001)	(0.001)	(0.001)	(0.002)	(0.001)	(0.001)	(0.001)	(0.001)	(0.001)	(0.001)
Loneliness	0.003***	0.002***	0.003***	0.002***	0.002***	0.0001	0.003***	0.003***	-0.001	0.003***	0.002***	0.001
	(0.052)	(0.065)	(0.034)	(0.050)	(0.029)	(0.090)	(0.043)	(0.084)	(0.118)	(0.026)	(0.031)	(0.075)
Sleep	0.002***	0.003***	0.004***	0.002***	0.001*	0.002*	0.003***	0.002***	0.003***	0.0005	0.002***	-0.002***
	(0.0005)	(0.0004)	(0.001)	(0.0004)	(0.0005)	(0.001)	(0.0005)	(0.0005)	(0.001)	(0.001)	(0.0004)	(0.001)
Frustration	0.001	0.001	0.001	0.002	0.003**	0.001	0.001	0.002	-0.001	0.001	0.002	-0.002
	(0.001)	(0.001)	(0.001)	(0.001)	(0.001)	(0.003)	(0.001)	(0.001)	(0.003)	(0.001)	(0.001)	(0.001)
Depression	-0.0005	-0.001**	-0.001**	-0.001	-0.001*	-0.001	-0.001**	-0.001	-0.002*	-0.001**	-0.001**	-0.001
	(0.001)	(0.001)	(0.001)	(0.0005)	(0.001)	(0.001)	(0.001)	(0.001)	(0.001)	(0.001)	(0.0005)	(0.001)
Anxiety	-0.078	-0.356*	-0.103	-0.219	-0.336***	0.031	0.175	0.075	0.032	0.021	-0.137	-0.311
	(0.170)	(0.210)	(0.111)	(0.162)	(0.092)	(0.289)	(0.138)	(0.280)	(0.387)	(0.084)	(0.101)	(0.247)
	Match Economic Activity											
Boredom	0.003	-0.005***	0.001	-0.005***	0.0003	0.0001	0.0003	0.003***	-0.002	0.001	-0.0001	-0.001*
	(0.002)	(0.001)	(0.001)	(0.001)	(0.0002)	(0.0002)	(0.0004)	(0.0005)	(0.002)	(0.001)	(0.0003)	(0.001)
Loneliness	0.003***	-0.001***	0.002***	-0.001***	0.0001	0.00003	0.0004**	0.001***	0.001	0.001***	0.00001	0.001***
	(0.001)	(0.0004)	(0.0003)	(0.0004)	(0.0001)	(0.0001)	(0.0001)	(0.0002)	(0.001)	(0.0003)	(0.0001)	(0.0003)
Sleep	-0.006***	-0.007***	-0.002***	-0.006***	-0.0004***	-0.001***	-0.002***	-0.001***	-0.006***	-0.004***	-0.002***	-0.004***
	(0.001)	(0.001)	(0.0005)	(0.001)	(0.0001)	(0.0001)	(0.0002)	(0.0003)	(0.001)	(0.0005)	(0.0002)	(0.0005)
Frustration	-0.006*	-0.004**	-0.001	-0.004**	0.001*	0.001*	-0.0003	-0.0002	-0.006*	-0.001	-0.001	-0.001
	(0.003)	(0.002)	(0.001)	(0.002)	(0.0003)	(0.0003)	(0.001)	(0.001)	(0.003)	(0.001)	(0.001)	(0.001)
Depression	0.002*	0.001	0.001	0.001	-0.0002	-0.0001	0.00001	0.0004	0.003**	-0.0004	-0.0002	-0.0004
	(0.001)	(0.001)	(0.001)	(0.001)	(0.0001)	(0.0001)	(0.0003)	(0.0004)	(0.001)	(0.001)	(0.0003)	(0.001)
Anxiety	-0.002	-0.002	-0.0001	-0.001	-0.0002	-0.0003	-0.001**	-0.0001	0.0001	-0.002**	-0.001*	-0.002*
	(0.002)	(0.001)	(0.001)	(0.001)	(0.0002)	(0.0002)	(0.0004)	(0.001)	(0.002)	(0.001)	(0.0004)	(0.001)
	Regression Statistics ( R2, F-test)											
Boredom	0.028	0.094	0.028	0.094	0.069	0.075	0.029	0.032	0.031	0.065	0.055	0.070
	8.629***	31.213***	8.666***	31.175***	22.162***	24.577***	8.981***	10.016***	9.526***	20.996***	17.652***	22.550***
Loneliness	0.112	0.195	0.123	0.196	0.141	0.144	0.114	0.111	0.124	0.143	0.159	0.147
	38.172***	72.963***	42.308***	73.247***	49.395***	50.663***	38.851***	37.544***	42.780***	50.077***	56.909***	51.817***
Sleep	0.049	0.055	0.044	0.054	0.051	0.052	0.068	0.057	0.046	0.066	0.079	0.068
	15.482***	17.539***	14.033***	17.330***	16.358***	16.681***	22.033***	18.251***	14.649***	21.396***	25.890***	22.009***
Frustration	0.006	0.011	0.006	0.009	0.008	0.007	0.008	0.007	0.005	0.005	0.008	0.005
	1.723	3.202**	1.785	2.726**	2.560*	2.260*	2.315*	2.054	1.601	1.440	2.430*	1.626
Depression	0.027	0.024	0.025	0.027	0.033	0.035	0.031	0.031	0.031	0.036	0.035	0.039
	8.394***	7.492***	7.823***	8.336***	10.255***	10.758***	9.638***	9.677***	9.623***	11.269***	10.899***	12.005***
Anxiety	0.011	0.010	0.004	0.008	0.004	0.008	0.015	0.014	0.005	0.012	0.015	0.021
	3.347**	3.062**	1247	2.416*	1.334	2.321*	4.498***	4.246***	1422	3.789**	4.723***	6.536***

Statistically significant at $$^{*}p<$$0.1; $$^{**}p<$$0.05; $$^{***}p<$$0.01

## Discussion

The pandemic has caused high levels of job uncertainty, which is characterized by job insecurity and unpredictable employment conditions. This job uncertainty can lead to significant psychological distress among employees [56–58], which is also affected by socioeconomic characteristics. As noted by [59], the economic and psychological effects varied among different income groups. Individuals in the low-income bracket, who were already facing economic hardships, experienced heightened psychological and financial distress. Conversely, the middle class, previously more secure, saw a rise in job insecurity accompanied by psychological issues. Those in the high-income group did not face economic difficulties but reported psychological impacts. Additionally, low-income earners expressed gratitude for the government’s financial aid, while middle-income earners felt that the financial support they received was insufficient.

Assuming that social attention to the topics presented in Table 1 proxies for the prevalence of the corresponding mental health disorders in the population, our findings suggest a strong association between different indicators of psychological well-being and the level of activity in different sectors of the economy.

For *Boredom*, *Loneliness*, and *Sleep*, we find that an increase in the business activities of small firms, controlling for the strength of NPIs, leads to a reduction in those feelings in the population. These results hold after controlling for the levels of official sectoral economic indexes provided by national economic authorities. Our results provide some support for an inverse relationship between the level of *Anxiety* and economic activity^12^. Although these results are not as strong as those for other measures of mental health well-being, they are in line with the fact that psychological distress associated with uncertainty in work can manifest itself in various forms, including anxiety and depression [61, 62]. For *Depression*, however, we find a positive association with the dynamism of the economic activity of the business verticals. This finding may seem contradictory at first, but it is important to note that working on-site during the pandemic can actually contribute to depression in certain individuals. The specific stressors and difficulties that come with on-site work during this time have been found to be associated with a higher likelihood of experiencing symptoms of depression, particularly for front-line health care workers and workers in essential services [63–65]. Interestingly, the business verticals for which there is a positive and significant relationship between depression and economic activity tend to be concentrated on on-site jobs, such as *Grocery and convenience stores, Manufacturing, Restaurants,* and *Retail*.

Aligning with the findings from recent multi-country studies [9, 10], our study underscores the pervasive impact of economic instability and job insecurity on mental health across different countries during the pandemic. These studies collectively highlight the need for robust support systems and interventions targeted at those affected by economic downturns to mitigate adverse mental health outcomes. Our research further enriches this dialogue by providing additional empirical evidence from Latin America, thus contributing to a broader understanding of the economic determinants of mental health during crisis periods [15, 16].

Finally, our results for *Sleep* are in line with the results in [66] that report that sleep problems are associated with higher levels of psychological distress. Similarly, [67] suggest that intolerance of uncertainty and perceived stress are critical factors in the relationship between COVID-19 uncertainty and sleep outcomes.

## Strengths and limitations

There are certain constraints to the analysis we conduct. To begin with, younger individuals are more likely to use Google Search in comparison to older age groups. Additionally, we do not have information about the sociodemographic characteristics of Google users. Lastly, GT searches may be subject to ambiguity: the interpretations of our search terms may change over time, either positively or negatively, without directly relating to the welfare of the Chilean population [17].

Despite the limitations noted above, current research supports the use of online health-related search data to monitor the progression, incidence, and spread of various diseases. A comprehensive systematic analysis reveals a notable increase in public queries related to mental health issues during the COVID-19 pandemic [68]. Wang et al. [35] suggest that Google Trends could serve as an innovative epidemiological method for mapping the prevalence of depression. In addition, the findings of Locatelli et al. [69] suggest the efficacy of Google Trends data in predicting coronavirus outbreaks.

When it comes to analyzing Business Activity Trends, it is important to note that the patterns observed in Facebook postings may not necessarily reflect concrete economic indicators such as sales or the overall size of a business. Differentiating between a decrease in activity due to specific events that affect business operations and normal fluctuations in business activity can be challenging. However, in the short term, this approach can be considered valid since the impact on business turnover is expected to be minimal. Additionally, it is crucial to acknowledge that businesses utilize Facebook in different ways, which can vary greatly depending on their country and industry. Therefore, we can expect diverse responses from businesses in different regions and sectors to disruptive events, and some of these variations may be influenced by the disruptive event itself.

Regardless, Facebook and other social networking applications appear to be effective tools for communities in dealing with disasters. A study conducted on small businesses in Boulder, Colorado, USA, after the floods revealed that these businesses actively utilized social media, specifically Facebook, during the various phases of disaster management, including preparedness, response, and recovery. The research findings highlight that small businesses often employ Facebook as a platform to share updates on road closures, cleanup operations, relief efforts, and the psychological impact of such incidents [39]. Eyre et al. [40] conducted a recent study that examined Facebook data to assess the recovery status of small businesses in urban areas following disasters. By analyzing case studies of earthquakes and hurricanes in Nepal, Puerto Rico, and Mexico, the researchers discovered that the posting patterns of small businesses on social media can offer real-time information about the progress of recovery in disaster-affected regions. Numerous additional research studies provide evidence of the significant role that social media played in emergency management, communication, and the dissemination of information [70–72].

While this study provides valuable insights into the correlation between small business activities and mental health in Latin America during the pandemic, there are several limitations that must be acknowledged. First, the reliance on Google Trends and Facebook Business Activity Trends as proxies for economic activity and mental well-being may not capture the full complexity of these dynamics. Additionally, the study’s focus on early pandemic stages might limit the applicability of findings to later phases or post-pandemic scenarios. These limitations suggest avenues for future research. Future studies could explore more direct measures of economic activity and mental health, including longitudinal data that spans various phases of health crises. Additionally, research could extend to other regions and economic contexts to validate and expand on our findings. Addressing these gaps can provide more nuanced public health insights and enhance preparedness for future health crises.

## Conclusions

In conclusion, this study underscores a significant correlation between the economic activities of small businesses and psychological well-being in Latin American countries during the initial stages of the pandemic. The findings suggest that government interventions aimed at supporting the economic resilience of SMEs can have direct implications for the mental health of the population. Therefore, we recommend that public policies focus on strengthening the financial stability of these businesses, fostering social support networks within the workplace, and integrating mental health services into work environments. These strategies can not only mitigate mental health challenges during future crises but also enhance public resilience in the face of increasingly complex health emergencies. Considering the high likelihood of economic and health challenges that may surpass those of the pandemic, it is crucial to adopt a proactive approach to public health interventions.

The worldwide COVID-19 pandemic has substantially affected mental health in numerous areas, with financial pressures being a major contributor [59]. Although there are differences between regions, common issues like joblessness, financial loss, and economic instability have consistently been linked to higher levels of anxiety, depression, and stress. Therefore, it is crucial to tackle these economic factors to alleviate the mental health crisis worsened by the pandemic.

## Data Availability

No datasets were generated or analysed during the current study.

## Footnotes

1 https://www.worldometers.info/coronavirus/

2 According to the OECDiLibrary, in 2022, SMEs constitute 65.3% of formal employment in Chile. In Colombia, these SMEs contribute to approximately 67% of employment and approximately 28% of GDP by the end of 2017. In Peru, in 2018, SMEs employ nearly 59.2% of the economically active population, while in Mexico they contribute to 71% of employment.

3 It should be noted that, compared with the other countries, Chile has been a high-income country for a considerable length of time. In any case, institutional, socioeconomic, and cultural characteristics undergo slow changes over time. Given the relatively brief period of our sample, these characteristics are captured by country fixed effects, and their differences between countries should not affect the estimated effects of our variables of interest.

4 https://data.humdata.org/dataset/facebook-business-activity-trends-during-covid19

5 The following abbreviations are used throughout the figures and tables in this manuscript: Grocery and convenience stores (GCS), Retail (Ret), Restaurants (Rest), Local events (Local), Professional services (Profe.Serv), Business and utility services (Bus), Home services (Home), Lifestyle services (Lifestyle), Travel (Trav), Manufacturing (MFG), Public good (Pub).

6 https://www.who.int/news/item/02-03-2022-covid-19-pandemic-triggers-25-increase-in-prevalence-of-anxiety-and-depression-worldwide

7 https://www.bcentral.cl/en/web/banco-central/area/statistics/imacec

8 In Argentina, the *EMAE* (https://www.indec.gob.ar/indec/web/Nivel4-Tema-3-9-48); in Colombia, the *IMAE* (https://www.banrep.gov.co/es; in Peru, the *PBI* (https://estadisticas.bcrp.gob.pe/estadisticas/series/mensuales/pbi-por-sectores); in Mexico, the *IGAE* (https://www.inegi.org.mx/temas/igae/).

9 Correlations are computed between the monthly average of the BAT and the corresponding economic activity index.

10 When interpreting the findings of the official economic index activities, it is important to exercise caution to prevent drawing conclusions that go beyond the intended goal of isolating the direct impact of BAT levels on the well-being of the population. This is because these indexes show less variability compared to the other included control variables.

11 As noted by [55], stricter enforcement of NPIs correlated with an increased likelihood of anxiety and depressive symptoms, a point that should be taken into account by governments in future crises.

12 Interestingly, during the outbreak, young people had a higher risk of anxiety than older people [60].
